# Refining Hypertension Surveillance to Account for Potentially Misclassified Cases

**DOI:** 10.1371/journal.pone.0119186

**Published:** 2015-03-24

**Authors:** Mingkai Peng, Guanmin Chen, Lisa M. Lix, Finlay A. McAlister, Karen Tu, Norm R. Campbell, Brenda R. Hemmelgarn, Lawrence W. Svenson, Hude Quan, Hypertension Outcomes Surveillance Team

**Affiliations:** 1 Department of Community Health Sciences, Faculty of Medicine, University of Calgary, Calgary, Alberta, Canada; 2 Department of Community Health Sciences, University of Manitoba, Winnipeg, Manitoba, Canada; 3 Department of Medicine, University of Alberta, Edmonton, Alberta, Canada; 4 Institute of Clinical Evaluative Sciences and Department of Family and Community Medicine, University of Toronto, Toronto, Ontario, Canada; 5 Department of Medicine, University of Calgary, Calgary, Alberta, Canada; 6 Surveillance and Assessment Branch, Alberta Health, Edmonton, Alberta, Canada; University of Perugia, ITALY

## Abstract

Administrative health data have been used in hypertension surveillance using the 1H2P method: the International Classification of Disease (ICD) hypertension diagnosis codes were recorded in at least 1 hospitalization or 2 physician claims within 2 year-period. Accumulation of false positive cases over time using the 1H2P method could result in the overestimation of hypertension prevalence. In this study, we developed and validated a new reclassification method to define hypertension cases using regularized logistic regression with the age, sex, hypertension and comorbidities in physician claims, and diagnosis of hypertension in hospital discharge data as independent variables. A Bayesian method was then used to adjust the prevalence estimated from the reclassification method. We evaluated the hypertension prevalence in data from Alberta, Canada using the currently accepted 1H2P method and these newly developed methods. The reclassification method with Bayesian adjustment produced similar prevalence estimates as the 1H2P method. This supports the continued use of the 1H2P method as a simple and practical way to conduct hypertension surveillance using administrative health data.

## Introduction

Hypertension is an important risk factor for renal, cerebrovascular, and cardiovascular diseases and can lead to premature mortality [1]. The Framingham Heart Study found that 91% of heart failure, 84% of strokes, and 70% of myocardial infarctions occurred in patients with hypertension [2,3,4,5]. An accurate estimate of hypertension prevalence is critical for evaluation of population based hypertension prevention, detection, and management programs.

Many countries have been developing hypertension surveillance programs. Using administrative health data and a previously validated cases definition based on International Classification of Disease (ICD) codes, the Public Health Agency of Canada (PHAC) reported that in Canada, national hypertension prevalence after age-standardization increased from 12.9% in 1999 to 19.6% in 2007 [6,7]. However, sometimes prevalence estimates from administrative health data tend to be higher than those from population-based cross-sectional surveys that actually measure blood pressures [8,9]. While some of this difference may be accounted for the fact that administrative health data captures virtually all patients while physical measures surveys usually only target community-dwelling individuals. It should also be acknowledged that underlying surveillance programs based on administrative health data assume that hypertension is a chronic and incurable disease. Once a patient has met the case definition in administrative health data, they are assumed to continue to have hypertension even if there are no further claims for this condition. However, some patients could have transient elevations of their blood pressure due to modifiable factors such as stress, high salt consumption, sedentary lifestyle, or use of nonsteroidal anti-inflammatory drugs (NSAID) medications—all of which are modifiable and resolution of them could result in a patient attaining normal blood pressure levels again. Prevalence could be overestimated once the number of false positive cases exceeds the number of false negative cases due to accumulation.

Administrative health data are collected for management purposes. Diagnoses or conditions in the data could be coded incorrectly or incompletely. Several studies improved the accuracy of specific case definitions in the data using statistical learning methods, such as regularized logistic regression, classification tree, and artificial neural network [10,11]. These methods determine condition status based on additional data features, such as demographic and clinical characteristics of patients.

Recently, a Bayesian method has been used to improve the accuracy of disease surveillance using administrative health data for osteoarthritis and systemic autoimmune rheumatic diseases [12,13]. Bayesian method provides a framework to incorporate prior information on sensitivities and specificities of cases definitions and prevalence of diseases in the study population [14]. Bayesian method adjusts for misclassified cases and take into account the variability issue of the case definition when applied in external populations.

We introduced two innovative methods to improve the accuracy of hypertension surveillance using the administrative health data. The first method was to develop and validate a new reclassification method to ascertain hypertension cases using a chart review dataset linked with administrative health data using the regularized logistic regression. The second method was to employ a Bayesian method to adjust the estimated prevalence. We compared hypertension across three methods, including traditional method used in Canada (i.e. one hospitalization or two claims with hypertension) and the two newly developed methods.

## Materials and Methods

### Chart review data

We extracted chart information for a random sample of 1565 patients from 28 general practitioners (GPs)/family physicians (FPs) and linked with administrative health data in Alberta, Canada [15], which had a population of approximately 3.65 million in 2011. We included fee-for-service GPs/FPs who practiced >2 days per week at their current locations between 1999 and 2001 or 2002 and 2004. Their patients were randomly selected based on the following criteria: ≥35 years of old, alive or did not migrate out of the province in the 2 year-period before the study year and ≥2 visits to a GP/FP with the study period. We excluded physicians who primarily practiced in walk-in clinics, community health centers, hospitals, emergency rooms or locum physicians. Diagnosis of hypertension in the chart was defined based on recorded blood pressure readings following the Canadian Hypertension Guidelines [16] or a physician-assigned diagnosis of hypertension in the notes. Patients with pregnancy-induced hypertension were excluded. The chart review data were used to develop the reclassification method for hypertension case identification.

### Administrative health data

Alberta has a universal single-payer health care system that covers all of the physician and hospital services and approximately 99% of provincial residents [17]. Alberta administrative health data include the population registry, the hospital discharge abstract (DAD), and physician billing claims. These data can be linked together using an anonymous personal identifier. The population registry includes the demographic and geographic information of residents, such as sex, age and registry status. DAD captures all patients discharged from hospitals in Alberta and each DAD record has up to 16 diagnoses coded using ICD, 9^th^ version, Clinical Modification (ICD-9-CM) before April 1, 2002 or up to 25 diagnoses using the ICD, 10^th^ version, Canadian enhanced version (ICD-10-CA) after March 31, 2002. Physician claims capture visits with physicians in the province and each claim has up to three diagnoses coded in ICD, 9th version (ICD-9). We extracted records with hypertension ICD diagnosis codes (ICD-9: 401.x, 402.x, 403.x, 404.x, or 405.x; ICD-10-CA: I10.x, I11.x, I12.x, I13.x, or I15.x) in DAD or physician claims data from the fiscal year of 1994 to 2009. We excluded pregnancy-induced hypertension and included patients aged 20 years or older. The observation period for each patient was defined up until date of death, date of moving out of Alberta, or March 31, 2009 (end of follow-up) using the population registry data.

### 1H2P method

A hypertension case definition, namely 1H2P method, was developed and validated in previous studies [15,18]. The 1H2P method is based on the following criteria: either 1 hospitalization or 2 physician claims within a 2 year-period with a hypertension ICD diagnosis code. Concretely, when a patient had a physician claim for hypertension, a second claim for hypertension within the two year-period was required in order for the patient to meet the case definition for the two-physician claim algorithm (and the index date for hypertension diagnosis was back dated to the time of the first claim). When a patient had one DAD record with hypertension ICD codes, the patient was assigned to have hypertension with the date of admission as the index date. Patients were assumed to have hypertension throughout follow-up period once they met this case definition.

### Reclassification method

To improve the accuracy of hypertension identification, a reclassification method was developed using regularized logistic regression with presence of hypertension in the chart review as the dependent variable. The regularization parameter was introduced in the estimation function for model parameters of logistic regression [19].

$$y=h_{\beta_{0},\;\beta }\left(x\right)=\frac{1}{1+e^{-\left(\beta_{0}+\beta^{T}x\right)}}$$ (1)

$$L\left(\beta_{0},\beta \right)=\overset{m}{\underset{i=1}{\sum }}\left[y_{i}\text{log}\left(h_{\beta_{0},\beta }\left(x_{i}\right)\right)+\left(1-y_{i}\right)\text{log}\left(1-h_{\beta_{0},\beta }\left(x_{i}\right)\right)\right]$$ (2)

$$J\left(\beta_{0},\beta \right)=L\left(\beta_{0},\beta \right)-\lambda \beta^{T}\beta$$ (3)

where *β*_0_ is the intercept term; *β* is the *n* x 1 vector of parameters in the model; *n* is the total number of independent variables; *m* is the total number of subjects in the dataset (sample size of chart review); *y* is the status of hypertension defined by chart review; *x* is the *n* x 1 vector of independent variables; *h*_*β*o_,*β*(*x*) is the logistic regression function; *L*(*β*_0_,*β*) is the log likelihood function for the logistic regression model; *λ* is the regularized term; *J*(*β*_0_,*β*) is the final function to be maximized to estimate the parameters of *β*_0_ and *β* in the logistic regression model. The introduction of *λ* helps to penalize the model with extreme parameter values and prevent the problem of over-fitting when the ratio of the number of subjects (*m*) to the number of independent variables (*n*) is small.

Independent variables included in the logistic regression model were age, sex, hypertension information from DAD and physician claims, Charlson and Elixhauser comorbidities [20,21]. Comorbidities were defined based on physician claims in 3 year-period using validated coding algorithms [22]. For example, the variables for an individual in 2001 were defined using information in physician claims from 1999 to 2001. We counted the number of claims for each comorbidity in each year. Diabetes with and without complications was combined as one variable. Variable for the AIDS/HIV was dropped due to zero cases in our sample. The comorbidities information in DAD were not used due to minor improvement in classification accuracy in preliminary analysis. Variables for hypertension were defined separately for the DAD and physician claims. We created a binary variable to flag the presence of diagnosis for hypertension defined by DAD in each fiscal year. For hypertension defined using physician claims, we created numerical variable through counting the number of claims for hypertension within each fiscal year. In total, there were 92 independent variables, including 1 numeric variable for age, 1 binary variable for sex, 1 numerical variable for hypertension in physician claims each year, 1 binary variable for hypertension in DAD each year, 84 numerical variables for comorbidities in physician claims. All the variables were normalized before model fitting process [19].

The performance of reclassification method depends on the choice of probability cutoff value and regularized parameter. We used the ten-fold cross validation with c-statistic as the measurement of fit to evaluate a set of regularized parameter values (0.01, 0.3, 0.1, 0.3, 1, 3, 10, and 30) and probability cut-off values (0.05 to 0.95, with an interval of 0.05). After finding the optimal parameters (λ = 1 and probability cutoff (P) = 0.25), we calculated the sensitivity, specificity, positive predictive value (PPV), negative predictive value (NPV) and kappa for the reclassification method. The 95% confidence intervals for the validity index were calculated using the bootstrap method [23]. The package of “LiblineaR” in R software [24] was used to fit the regularized logistic regression.

### Bayesian adjustment for results from the reclassification method

Reclassification method could misclassify cases and its model performance has potential variations in external populations. Bayesian method was used to adjust for the prevalence estimated from the reclassification method.

Prior distributions of sensitivity and specificity for the population of 20 year or older and then for population aged 65 years or older were derived using the bootstrap method on chart review data with an age-weighted sampling strategy. The chart data covered the patients aged 35 years or older (Table 1). We used the patients at 35 years old as the sampling replacement in bootstrap method for patients aged from 20 to 34 years. Age distribution was right-skewed and the age distribution in fiscal year 2003 (the midpoint of this study period) was used as the reference for sampling. Beta distribution was used to specify the prior distributions for sensitivity and specificity [14]. The parameters for the beta distribution were selected by matching the 50^th^ and 75^th^ percentiles for the empirical distributions of sensitivity and specificity. The prevalence of hypertension was assumed to be non-informative and followed the beta distribution with parameter of 1 and 1.

**Table 1:** Characteristics of patients in the chart review data.

	Hypertensive^a^ cases (N = 396)		Non-hypertensive cases (N = 1169)		
	N	%	N	%	P value
Age					<0.001
35–54 years	125	31.6	906	77.5	
55–64 years	92	23.2	146	12.5	
65+ years	179	45.2	117	10.0	
Sex					
Male	175	44.2	366	31.3	<0.001
Comorbidities^b^ in physician claim					
Diabetes	44	11.1	22	1.9	<0.001
Myocardial infarction	29	7.3	7	0.6	<0.001
Cerebrovascular disease	28	7.1	17	1.5	<0.001
Congestive heart failure	34	8.6	8	0.7	<0.001
Rheumatic disease	7	1.8	11	0.9	0.252
Dementia	6	1.5	4	0.3	0.026
Peripheral vascular disease	9	2.3	6	0.5	0.004
Paralysis	0	0	2	0.2	0.635
Chronic pulmonary disease	51	12.9	140	12.0	0.645
Renal failure	14	3.5	5	0.4	<0.001
Pulmonary circulation disorders	9	2.3	8	0.7	0.011
Metastatic cancer	0	0	5	0.4	0.344
Cardiac arrhythmias	36	9.1	23	2.0	<0.001
Peptic ulcer disease	7	1.8	16	1.4	0.621
Valvular disease	13	3.3	9	0.8	0.002
Hypothyroidism	19	4.8	77	6.6	0.226
Lymphoma	2	0.5	5	0.4	1
Solid tumor without metastasis	32	8.1	36	3.1	<0.001
Anemia	6	1.5	7	0.6	0.098
Coagulopathy	11	2.8	12	1.0	0.018
Fluid and electrolyte disorder	16	4	27	2.3	0.085
Weight loss	4	1	5	0.4	0.255
Obesity	10	2.5	30	2.6	1
Alcohol abuse	2	0.5	10	0.9	0.542
Drug abuse	17	4.3	37	3.2	0.328
Psychoses	4	1	9	0.8	0.747
Depression	93	23.5	294	25.1	0.537
Liver disease	8	2	13	1.1	0.195

^a^ Hypertension status was defined based on the chart review.

^b^ Diagnosis information of hypertension and other comorbidities were retrieved at or up to 2 years before the study fiscal year.

The Gibbs sampling technique with 50,000 iterations and a burn-in period of 10,000 iterations was used to derive samples for the marginal posterior density for the parameters including prevalence. Convergence was assessed by evaluating the trace plots for each parameter [25]. The median value of the posterior distribution was used as the point estimate for the prevalence. R program code used to conduct the Bayesian adjustment is in S1 Appendix.

### Estimation of hypertension prevalence using administrative health data

**Fig 1:**
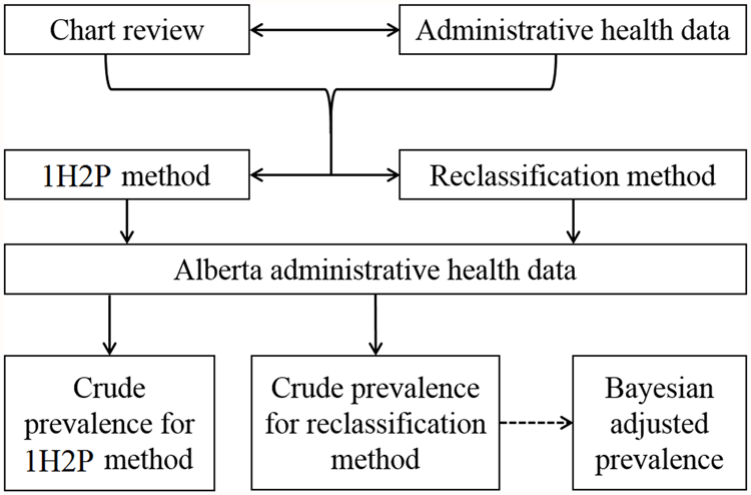
The diagram of methods developed and used in the study. *1H2P method: at least 1 hospitalization or 2 physician claims within two year-period for hypertension coded in International Classification of Diseases.

Using the administrative health data, we defined patients with hypertension using 1H2P and reclassification methods, respectively. Reclassification method was applied at each fiscal year to identify the hypertension cases in the study population without carrying forward the case definitions into the following year. Bayesian method was then used to adjust the results reported from the reclassification (see Fig. 1).

Hypertension prevalence at two age groups (20 years or older and 65 years or older) were reported for (1) the 1H2P method, and the reclassification method (2) with and (3) without Bayesian adjustment. We estimated annual age-specific prevalence from fiscal year 1996 to 2009 by dividing the number of hypertension cases in each fiscal year by the population estimate from the Alberta registry data in the corresponding year.

This study was approved by The Conjoint Health Research Ethics Board (CHREB), University of Calgary. Participating physicians provided written informed consent. All the records were anonymized.

## Results

Patients with hypertension were older than patients without hypertension (45.2% vs. 10% for age 65 years or older, see Table 1). Hypertension related comorbidities were more prevalent among patients with hypertension than patients without hypertension (11.1% vs. 1.9% for diabetes, 7.3% vs.0.6% for myocardial infarction, and 8.6% vs. 0.7% for congestive heart failure).

With the probability cutoff selected, the reclassification method had a higher sensitivity and kappa statistic but lower specificity than the 1H2P method (for age ≥35 years, sensitivity: 86.1% vs.73.7%, kappa: 0.787 vs. 0.735, specificity: 93.8% vs. 96.2%, see Table 2). The 1H2P method resulted in 45 false positive cases and 104 false negative cases with the chart-reviewed data as the gold standard, while the reclassification method resulted in 73 false positive cases and 55 false negative cases. Validity of the reclassification method varied by age. Sensitivity increased with age (86.1% for age ≥ 35 vs. 92.9% for age ≥65) while the specificity decreased (93.8% for age ≥ 35 vs. 70.8% for age ≥65). We recalculated statistics of validity based on age-weighted sampling to take into account the difference of age composition between the chart review data and Alberta administrative health data (see Table 3 for parameters).

**Table 2:** Validity of administrative data in defining hypertension using the 1H2P and reclassification methods.

	1H2P method		Reclassification method^a^	
	Mean	95% CI	Mean	95% CI
Data for people aged 35 years or older				
Sensitivity, %	73.7	69.4, 77.9	86.1	82.7, 89.2
Specificity, %	96.2	95.0, 97.2	93.8	92.4, 95.2
PPV, %	86.7	83.0, 90.3	82.4	78.7, 86.3
NPV, %	91.5	90.4, 93.0	95.2	94.0, 96.4
Kappa	0.735	0.698, 0.775	0.787	0.755, 0.821
Data for people aged 65 years or older				
Sensitivity, %	79.6	73.3, 85.2	92.9	88.9, 96.2
Specificity, %	86.7	80.0, 92.4	70.8	62.5, 79.1
PPV, %	90.0	85.1, 94.3	82.8	77.4, 87.6
NPV, %	73.7	66.1, 80.5	86.8	80.0, 93.0
Kappa	0.642	0.551, 0.728	0.656	0.566, 0.741

1H2P, two physician claims for hypertension within two year-period or one recording of hypertension in hospital discharge data; CI, confidence interval; PPV, positive predictive value; NPV, negative predictive value.

^a^ The validity reported here were calculated based on the probability cutoff of 0.25. It could change with the choice of difference probability cutoff value.

**Table 3:** Prior distribution of sensitivity and specificity for the reclassification method at two age groups: 20 years or older and 65 years or older.

	Median, %	Interquartile range, %	α	β
Age group, 20 years or older				
Sensitivity	89.1	88.2, 90.0	471.7	57.8
Specificity	90.9	90.2, 91.5	827.6	83.5
Age group, 65 years or older				
Sensitivity	93.6	92.2, 94.8	152.1	10.7
Specificity	69.7	66.6, 72.6	76.2	33.4

α and β refer to the parameters of the beta distribution and were determined by matching the 50^th^ and 75^th^ percentiles of the sensitivity and specificity distribution. The sensitivity and specificity distribution was constructed using the age-weighted bootstrap method.

**Fig 2:**
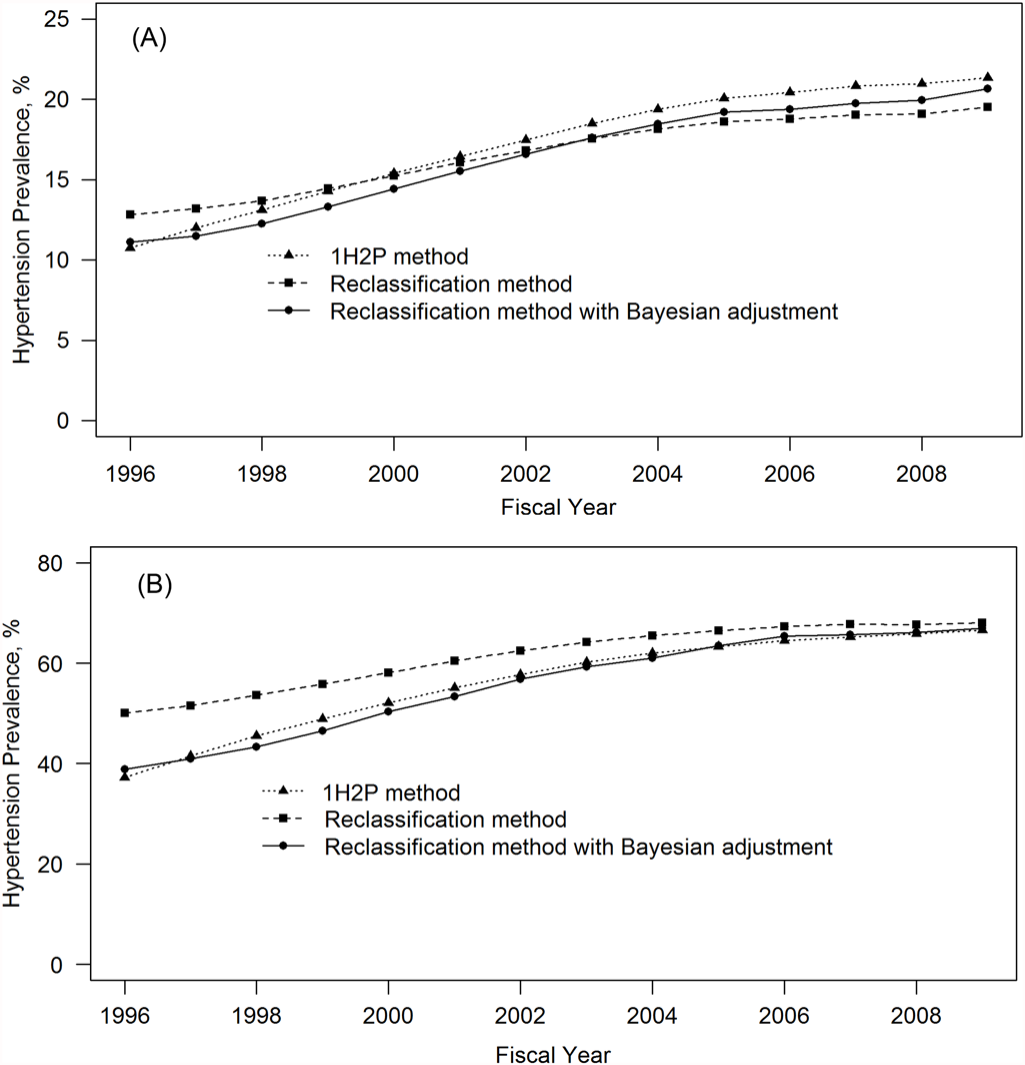
Crude prevalence of hypertension among A) adult aged 20 years and older and B) adults aged 65 years or older in Alberta, Canada from 1996 to 2009.

We calculated hypertension based on three methods (See Fig. 2). For age 20 years or older (Fig. 2A), prevalence increased from 10.7% in 1996 to 21.3% in 2009 (absolute difference: 10.6%) for the 1H2P method, from 12.8% to 19.5% (absolute difference: 6.7%) for the reclassification method, and from 11.1% to 20.7% (absolute difference: 9.6%) for the reclassification method with Bayesian adjustment. The prevalence from the 1H2P method was consistently higher (around 1%) than that from the reclassification with Bayesian adjustment.

For age 65 years or older, hypertension prevalence estimated from the reclassification method was consistently higher than prevalence from the 1H2P method (see Fig. 2B). After Bayesian adjustment, the reclassification method had the similar prevalence as the 1H2P method.

## Discussion

We found that the reclassification method using multiple data features from administrative health data improved sensitivity for identifying hypertension cases with slight loss of specificity. The Bayesian method effectively adjusted the prevalence by incorporating prior knowledge on distribution of sensitivity and specificity. The reclassification method with Bayesian adjustment produced similar prevalence estimates as the 1H2P method.

The reclassification method has been used for disease surveillance [10]. It is based on the assumption that all positive cases are true cases. In our study, compared with 1H2P method, the reclassification method identified more hypertension cases and more false positive cases. Importantly we found that age affects the sensitivity and specificity of the reclassification method, and the validity of prevalence from the reclassification method depends on the magnitude of hypertension prevalence, which is affected by population age composition. For example, in the age group of 65 years or older with high prevalence of hypertension, overestimation of hypertension prevalence from the reclassification method occurred. More false positive cases than false negative cases were generated due to low specificity (69.7%) and high sensitivity (93.6%). The Bayesian adjustment takes into account the imperfection and uncertainty of sensitivity and specificity in the classification process and produced adjusted prevalence estimates as expected. Bayesian method can also be used to adjust the prevalence estimated from the 1H2P method if cases were not accumulated with years of follow-up.

The 1H2P method is a simple and practical surveillance method and has high level of validity in Canadian administrative health data [6,7,8,9,15,18,26]. Our study found that the 1H2P method produced similar estimates of hypertension prevalence as the Bayesian adjusted prevalence. Although concerns have been raised about the potential overestimation arising from accumulation of false positive cases using the 1H2P method, it should be acknowledged that this could be balanced with false negative cases related to physician billing practice. Physicians are more likely to report hypertension when billing for patients with uncomplicated essential hypertension than those with chronic complications of hypertension. Physicians rarely report more than one condition in their billings although there are three ICD coding fields for diagnosis available in the Alberta administrative health data. Only about 5% of physician claims in Alberta had more than one ICD diagnosis code. Because payment is not determined by conditions and number of diagnoses (i.e. case-mix), patients with complications of hypertension are potentially misclassified as non-hypertension cases, generating false negative cases. It should be noticed that accumulation of cases with follow-up could increase sensitivity while decreasing specificity for the 1H2P method.

Performance of the 1H2P method depends on administrative health data quality. Quality is strongly related to physician documentation, coding guidelines, coder training and physician payment models [27,28]. For example, some provinces in Canada only have 1 ICD field for diagnosis in their physician billing claims. PPV and NPV also depend on prevalence of the condition. In our study, hypertension prevalence increases with age and NPV for 1H2P method was 91.5% for age ≥35 and 73.7% for age ≥65. These factors could affect the number of false positive cases and the number of false negative cases, which could lead to invalidity of the 1H2P method. Therefore, our results should be generalized with caution to other populations.

Our study has limitations. First, the reclassification method included a limited number of variables, such as age, sex, and comorbidities. Inclusion of other factors such as drug prescriptions and blood pressure measurements could provide complementary information for hypertension diagnosis code in clinical setting and improve reclassification accuracy [29]. High prevalence of hypertension and high validity for the 1H2P method left limited margin of improvement for the reclassification method. Validity of reclassification method depends on the choice of probability cutoff value. The probability cutoff value for hypertension reclassification can’t be generalized to diseases without testing. Second, without actual blood pressure measurement, we do not have a true ‘gold standard’ to determine which method generates the prevalence closest to the ‘truth’. It is unclear when and how the balance between accumulated false positive and false negative cases is achieved. Third, we analyzed the data from a single province. Thus generalizability of our findings needs to be assessed by replication in administrative health data from other jurisdictions.

In summary, administrative health data is a cost-effective source to conduct population based hypertension surveillance. Our comparison of 3 different methods of generating hypertension prevalence estimates revealed little difference between methods. As the 1H2P method is the simplest method to conduct hypertension surveillance in administrative health data and the 2 more complicated methods do not provide substantively different prevalence estimates, we believe continued use of the 1H2P method is appropriate. For conditions with high validity, there is limited margin left for improvement using statistical methods. The statistical methods could be used to improve the surveillance for conditions with low sensitivity or specificity of case definition. Conditions with low prevalence are likely to have low PPV and high NPV. The statistical methods is also helpful for rare diseases or low NPV/PPV.

## Supporting Information

S1 Appendix (DOCX)
