# Supplementary material for: Refining Hypertension Surveillance to Account for Potentially Misclassified Cases
Source: PLoS One. 2015 Mar 24;10(3):e0119186. doi: 10.1371/journal.pone.0119186 (PMC4372561; doi:10.1371/journal.pone.0119186)
Supplement: S1 Appendix — (DOCX) [file pone.0119186.s001.docx]

**Appendix:**

**R function for Bayesian adjustment on prevalence estimates based on the prior distribution of sensitivity and specificity of case definition and test results in a population. The function was written based on the paper of Joseph et al. 1995.**

prevalence.bayes <- function(a,b,pi_initial,S_initial,C_initial,Numloop, Pi_s1,Pi_s2,S_s1,S_s2,C_s1,C_s2){

## a: The number of positive cases in the test

## b: The number of negative cases in the test

## pi_initial: initial value for the prevalence

## S_initial: initial value for the sensitivity of test

## C_initial: initial value for the specificity of test

## Numloop: the number of sample

## Pi_s1 and Pi_s2: the parameter of prior beta distribution on prevalence

## S_s1,S_s2: the parameters of prior beta distribution for sensitivity

## C_s1,C_s2: the parameter of prior beta distribution for the specificity

Y1 <- NULL

Y2 <- NULL

S <- NULL

Pi <- NULL

C <- NULL

Iteration <- NULL

for (i in 1:Numloop){

Iteration[i] <- i

prob1 <- pi_initial*S_initial/(pi_initial*S_initial+(1-pi_initial)*(1-C_initial))

Y1[i] <- sum(rbinom(a,1, prob1))

prob2 <- pi_initial*(1-S_initial)/(pi_initial*(1-S_initial)+(1-pi_initial)*C_initial)

Y2[i] <- sum(rbinom(b,1, prob2))

shape1_Pi <- Y1[i]+Y2[i]+Pi_s1

shape2_Pi <- a+b-Y1[i]-Y2[i]+Pi_s2

Pi[i] <- rbeta(1, shape1_Pi, shape2_Pi)

pi_initial <- Pi[i]

shape1_S <- Y1[i]+S_s1

shape2_S <- Y2[i]+S_s2

S[i] <- rbeta(1, shape1_S, shape2_S)

S_initial <- S[i]

shape1_C <- b-Y2[i]+C_s1

shape2_C <- a-Y1[i]+C_s2

C[i] <- rbeta(1, shape1_C, shape2_C)

C_initial <- C[i]}

output <- cbind(Iteration, Pi,S,C)

return(output)}

## Example:

## It is assumed that the population size is 1000 with known prevalence of disease and the sensitivity and specificity of test is known and fixed.

prevalence <- 0.55

Sample <- 1000

Sensitivity <- 0.81

Specificity <- 0.95

a <- Sample*prevalence*Sensitivity + Sample*(1-prevalence)*(1-Specificity)

b <- Sample*prevalence*(1-Sensitivity) + Sample*(1-prevalence)*Specificity

### Bayesian adjustment

pi_initial <- 0.10

S_initial <- 0.5

C_initial <- 0.5

Numloop <- 20000

Pi_s1 <- 1

Pi_s2 <- 1

S_s1 <- 20.22

S_s2 <- 5.30

C_s1 <- 241.36

C_s2 <- 13.42

Results <- prevalence.bayes(a,b,pi_initial,S_initial,C_initial,Numloop,Pi_s1,Pi_s2,S_s1,S_s2,C_s1,C_s2)

## Results

plot(Results[,1],Results[,2],type="l", xlab="Iteration",ylab="Prevalence")

abline(h=prevalence,col="red")

plot(Results[,1],Results[,3],type="l", xlab="Iteration",ylab="Prevalence",ylim=c(0,1))

abline(h=Sensitivity,col="red")

plot(Results[,1],Results[,4],type="l", xlab="Iteration",ylab="Prevalence",ylim=c(0,1))

abline(h=Specificity,col="red")

cat('median and 95% confidence interval for prevalence',

quantile(Results[2000:20000, 2],c(0.5, 0.025, 0.975)),"\n")

cat('median and 95% confidence interval for sensitivity',

quantile(Results[2000:20000, 3],c(0.5, 0.025, 0.975)),"\n")

cat('median and 95% confidence interval for specificity',

quantile(Results[2000:20000, 4],c(0.5, 0.025, 0.975)),"\n")
